# Effects of Mindfulness-Based Interventions on Promoting Athletic Performance and Related Factors among Athletes: A Systematic Review and Meta-Analysis of Randomized Controlled Trial

**DOI:** 10.3390/ijerph20032038

**Published:** 2023-01-22

**Authors:** Yan Wang, Si-Man Lei, Jingjing Fan

**Affiliations:** 1 Faculty of Education, University of Macau, Taipa, Macau SAR 999078, China; 2 Department of Physical Education, Shanghai Sports University, Shanghai 200438, China

**Keywords:** mindfulness, athletic performance, athletes, randomized controlled trial, psychological components, mental health

## Abstract

In recent years, mindfulness-based interventions (MBIs) have been widely applied in competition sports with respect to athletic performance and mental health promotion, whereas evidence of randomized controlled trials (RCTs) has not been well summarized. Therefore, this study aimed to systematically review and meta-analyze the existing evidence on the effects of MBIs on improving athletic performance, mindfulness level, mindfulness-related psychological components (e.g., acceptance, self-compassion, flow), and mental health (e.g., burnout, stress, psychological well-being) among athletes. Following the PRISMA guidelines, a literature search was implemented on five electronic databases (Web of Science, PubMed, Scopus, ProQuest, and ScienceDirect) and relevant review papers. The article selection, risk of bias assessment, and data extraction were performed by two investigators independently. The standardized mean difference (SMD) was calculated to evaluate the effects of interventions using the random effect model. Among the 1897 original hits, thirty-two eligible RCT studies were included in the systematic review, of which seven were involved in the meta-analysis. The results showed that MBIs were effective in promoting athletes’ athletic performances (by narrative synthesis), mindfulness-level (n = 3; SMD = 0.50, 95% CI = [0.17, 0.83]; I^2^ = 45%, *p* = 0.16), and mindfulness-related psychological components (n = 5; SMD = 0.81, 95% CI = [0.53, 1.10], I^2^ = 77%, *p* =0.001), while no significant intervention effects were found on the mental health of athletes (n = 4; SMD = −0.03, 95% CI = [−0.35, 0.29], I^2^ = 89%, *p* < 0.001). Our findings preliminarily support the potential effectiveness of MBIs, whereas more high-quality RCTs were needed in the future.

## 1. Introduction

In competitive sports, pursuing optimal athletic performance is the ultimate goal for athletes [1]. During past decades, sport psychologists have developed diverse psychological training approaches, endeavoring to help athletes promote their competitive performance [2]. As a traditional stream of psychological training, cognitive behavioral therapies (CBTs) have been widely applied and shown effectiveness in the promotion of athletic performance among athletes [3]. CBT emphasizes the self-control of internal states through the training of psychological skills, with a view of improving motor performance [3,4]. However, an increasing group of evidence has shown that negative effects might be caused by controlling, eliminating, or suppressing athletes’ internal states [5]. Some studies have also indicated that goal-oriented behaviors might be replaced by excessive cognitive activities [5].

To address the limitations of traditional CBTs, mindfulness-based interventions (MBIs) have raised increasing concerns, which have shown a great potential for promoting athletes’ competitive performances as an alternative of traditional psychological training approaches [6]. The term of mindfulness is derived from Eastern Zen [7], which is defined as non-judgmental, conscious, purposeful focus, understanding, and acceptance of the things around one in the present moment [8]. In contrast to the traditional behavioral therapies, mindfulness emphasized that individuals should be aware of and experience the internal and external thoughts and feelings without any judgments [9]. In the sports domain, mindfulness has been used to cultivate athletes’ abilities to accept and realize, to inspire athletes to effectively control their attention and improve their body perception ability [8,9,10]. Mindfulness underlines the process from perceiving to experiencing the constant changes of current things and one’s own emotions without the inhibition and control of undesirable responses in order to remove the emotional state of pervious worries, avoid performance failure under pressure, and adjust the mental state of athletes [11]. Mindfulness provides a potential not only to improve performance but also to decrease the risks of mental health problems [11].

With increasing understandings of the mechanisms of mindfulness with different outcomes, a series of mindfulness training approaches have been developed, such as Mindfulness-Acceptance-Commitment (MAC) training, Mindfulness-Based Stress Reduction (MBSR) training, and Mindfulness-based Cognitive Therapy (MBCT) [12,13,14,15]. Mindfulness has shown to be positively associated with a wide range of indicators among athletes, including greater athletic performance, better mindfulness, and related psychological components (e.g., acceptance, flow, and psychological flexibility) and lower risks of mental health problems (e.g., stress, anxiety, depression, and burnout) [9,11,12,13,14,15]. Two previous review and meta-analysis studies compiling evidence from diverse types of studies (e.g., RCT, case study, and observational) provided preliminary support for the effectiveness of mindfulness-based interventions (MBIs) on improving athletes’ sports performance [9,12,16]. Furthermore, previous review papers have also examined the effects of MBIs on the mindfulness-related psychological components (i.e., flow) and mental health among specific athlete samples (e.g., elite athletes and long-distance runners) [17,18,19]. However, none of previous review studies focused only on the evidence from RCTs (the rigorous study design), which may be due to the limited numbers of studies. In addition, the above review studies did not provide a comprehensive overview of the effectiveness of MBIs on a wide range of outcomes (i.e., performance, psychological components, and mental health) targeting all types of athletes (e.g., elite, collegiate, and retired).

Therefore, the present study aimed to systemically review and meta-analyze existing RCT evidence on the effects of MBIs in improving athletic performance, mindfulness, and mindfulness-related psychological components and mental health among athletes. It is expected that the research findings may contribute to future mindfulness research, interventions, and policymaking, with the aim of promoting sport performance and health among athletes.

## 2. Materials and Methods

### 2.1. Search Strategy and Study Selection

The current systematic review and meta-analysis were conducted following the Preferred Reporting Items for Systematic Review and Meta-Analyses (PRISMA) guidelines (Supplementary Materials); the review protocol has been prospectively registered on PROSPERO (CRD42022299940). Based on a predefined literature search strategy, the following five electronic databases were searched using Boolean logic’s multi-filed search format with no limit to the date range: Web of Science, PubMed, Scopus, ProQuest, and ScienceDirect. In addition, the reference lists of relevant review papers were also checked. We combined three groups of keywords in the search: (1) mindfulness; (2) sport* OR athlete* OR player* OR athletic* OR exercise* OR training; and (3) performance OR achievement. The literature search was limited to human participants and the publication language was limited to English.

Following the Population, Intervention, Comparison, Outcome, and Study design (PICOS) principles, the study selection criteria included:

*Population:* any types of athletes without limits to the demographics and sports levels (e.g., collegiate, recreational/amateur, professional, elite, retired, or handicapped) were included.

*Intervention:* any types of mindfulness-based interventions (MBI) without limits to the training types and components (e.g., traditional Mindfulness-Acceptance-Commitment intervention, Mindfulness-Based Stress Reduction intervention, mindfulness-based yoga, or mindfulness-based cognitive therapy), frequency, and duration were included.

*Comparison:* any control conditions without mindfulness components (e.g., non-treatment control, waiting-list control, and active control using CBT) were eligible.

*Outcome:* objectively or subjectively measured athletic performance was the primary outcome and secondary outcomes included the mindfulness level, mindfulness-related psychological components (e.g., acceptance, self-compassion, psychological flexibility, flow, and ruminative response) and mental health indicators (e.g., anxiety, stress, and depression).

*Study design:* due to the large number of studies found in the literature search after protocol submission, we decided to limit our analysis to randomized controlled trials (RCTs) as this type of study provided the highest standard of evidence. All the RCT designs were included, while quasi-experimental, observational, and case studies were excluded.

In addition, book chapters, editorial letters, commentaries, conference proceedings, qualitative articles, and review articles were not eligible for inclusion.

All the initially searched articles were exported into the Mendeley to remove the duplications. Two investigators (W.Y. and F.J.J.) independently screened the titles and abstracts of all the remaining articles, where all clearly irrelevant articles were deleted. Afterwards, the full texts of all the identified articles were checked for eligibility by two investigators independently according to the aforementioned inclusion criteria. During the review process, any disagreements were addressed by consensus or by involving a third investigator (SML).

### 2.2. Data Extraction

A standardized form was used to extract data from the full texts of eligible articles by one investigator (WY). A second investigator (FJJ) checked the accuracy of the data extraction. All the information including authors, publication year, study design, participants, sample size, mean age, intervention duration, frequency, measures, and main findings were extracted to the Excel sheet for the following narrative synthesis and meta-analysis.

### 2.3. Risk of Bias Assessment

The study quality of included studies was evaluated using the Cochrane risk-of-bias tool in Review Manager software (RevMan, version 5.4, Cochrane Collaboration, Oxford, UK). Seven aspects of biases, including random sequence generation (selection bias), concealment of allocation sequence (selection bias), blinding of participants and personnel (performance bias), blinding of outcome assessment (detection bias), incomplete outcome data (attrition bias), selective outcome reporting (reporting bias), and others, were assessed. The overall quality of the study was categorized as low risk (“low risk” in all aspects), unclear risk (at least one aspect with “some concerns of biases”), and high risk (at least one aspect with “high risk” or several aspects with “some concerns”). The study quality was evaluated by two investigators (W.Y. and F.J.J.) independently and any discrepancies were resolved by consensus or confirmation of a third investigator (SML).

### 2.4. Statistical Analysis

The meta-analyses were implemented using RevMan 5.4 (Cochrane Collaboration, Oxford, UK) if at least three studies presented the same exposure with adequate information for the effect size estimates [20,21,22]. The standardized mean difference (SMD) with 95% confidence intervals (CI) was extracted from the pre- and post-intervention. For studies that reported other effect sizes (e.g., odds ratio and Cohen’s *f*^2^), the SMDs were calculated using a conversion spreadsheet [22,23]. Pooled effect sizes were estimated using a random effects model and were presented in forest plots. The heterogeneity was assessed using the I^2^, with I^2^ values of 25%, 50%, and 75% indicating low, medium, and high heterogeneity, respectively [24]. The publication bias was evaluated using funnel plots and Egger’s regression tests [25,26]. The subgroup analyses were implemented on study quality and demographics in case the number of included samples is no less than 10 [22,27,28]. The statistical significance level was set as *p* < 0.05 for effect sizes estimation and *p* < 0.10 for heterogeneity and publication bias assessments [27,28].

## 3. Results

### 3.1. Study Characteristics

**Figure 1:**
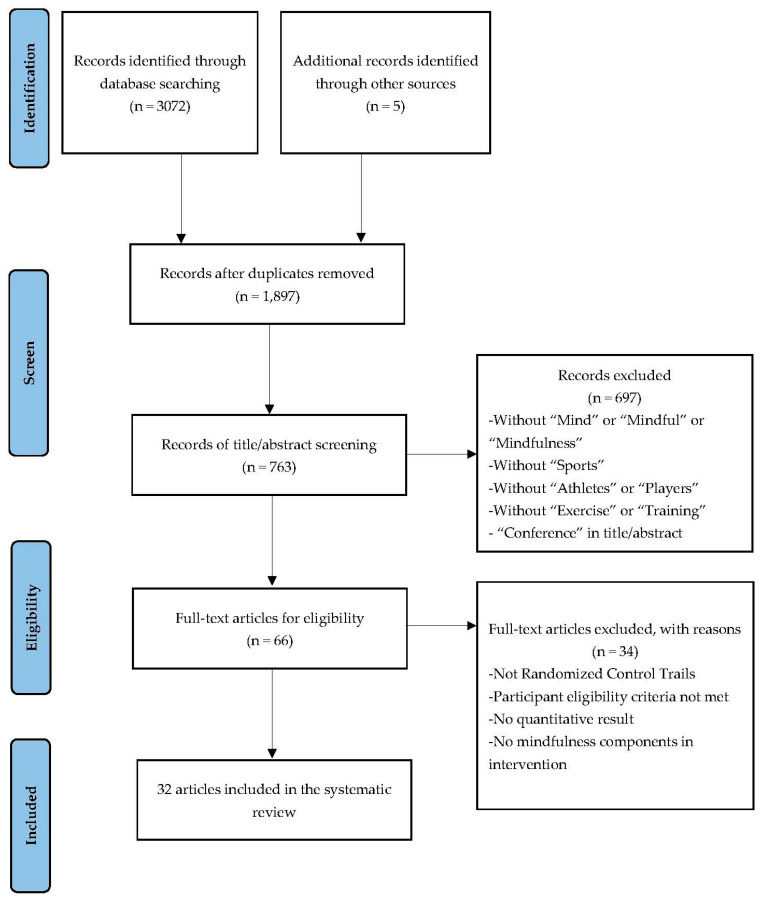
PRISMA flow chart was used to identify of the include studies.

Figure 1 shows the PRISMA flow diagram. Initially, we retrieved 3072 records from the databases search and identified an additional five articles from other sources. After the duplicates were removed, 1897 articles were screened by two investigators at the title and abstract level and the full texts of 66 potential articles were assessed. Finally, we included 32 articles after removing ineligible articles.

The feature coding results show that the studies included in this review came from a wide range of countries and territories: seven studies reported data from Iran; five from the USA; three each from Switzerland, Brazil, and Sweden; two each from Portugal, India, Australia, China, and Germany; and one from Norway.

The characteristics of studies included in this review are presented in Table 1. All these 32 studies were RCT studies. A total sample of 1788 athletes from a variety of sports were included, with an average age of 23.67 years. The athletes were involved in basketball, football, shooting, volleyball, wushu, cycling, track and field, and other sports. There was also a range of athletic experience from amateur to elite international athletes, with most studies including athletes competing at university level or higher. All the included trails were published after 2009, ranging from 2010 to 2022. The average intervention period was 6.3 weeks and the intervention training period was different in different programs. The intervention programs mainly include MAC, MBCT, MBSR, brief mindfulness training, and other mindfulness-based intervention types.

**Figure 2:**
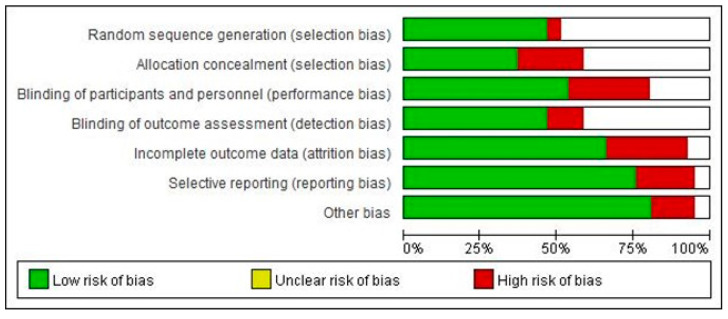
Risk of bias assessment for the included studies.

For the risk of bias assessment, 71% of the included studies had a low risk of bias, while six studies (29%) were rated as a high risk of bias. Most studies showed a high risk of bias with respect to the allocation concealment (22%), blinding of participants and personnel (29%), incomplete outcome data (29%), and selective reporting (19%) (Figure 2).

### 3.2. Effects of Mindfulness-Based Interventions on Athletic Performance among Athletes

Consistent results were observed in the included studies, showing significant improvements in sport performance following MBIs. Specifically, a randomized controlled trial [29] examined the effects of a 15 min mindfulness intervention on basketball players’ athletic performance under pressure. The results showed that 15 min mindfulness intervention was effective in promoting participants’ first free-throw performance under a stressful setting compared to the control condition. In another study [30], the results found that participants in the intervention group (n = 18) receiving a 15 min mindfulness treatment had a small-to-moderate increase (Cohen’s d = 0.48) in free-throw performance compared with the control group (n = 18). Another RCT study [31] which examined the effectiveness of an eight-session MAC method on improving performance and sports competition anxiety in athletes found that MAC was effective in improving athletic performance compared to the control group. An American research group investigated the effectiveness of the MAC approach compared to traditional psychological skills training (PST) for mental health and sport performance [13]. The results indicated that the MAC group outperformed the PST group on the outcomes. There was also a significant within-group effect, where the MAC participants experienced significant increases in sport performance from pre-intervention to post-intervention (*p* = 0.04). In another study [32], compared with the PST group (n = 33), the MAC group (n = 36) reported higher levels of training performance (β = −0.10, 95% CI = [−0.14, −0.08] among elite athletes. In addition, a pilot study of MBSoccerP [39] explored the potential role of mindfulness and other psychological factors on flow and elite performance in soccer athletes. The results showed that after receiving eight 1.5–2 h weekly sessions of MBSoccerP, mindfulness mediated the peak performance of elite soccer players. In a Swiss study [33], researchers tested elite athletes (n = 133) from twenty-three different sports and the results indicated that the trait mindfulness was related to fewer performance worries and prevented the remaining worries from influencing athletes’ behavior, thereby helping them to perform better. As for the two studies of MMT, one of them estimated the effect of MMT on shooting performance in elite shooters (n = 96) and was assessed using a pre-and-post-test approach [34]. Compared to the control, the results of the experimental group showed that MMT intervention decreased pre-competition stress and enhanced shooting performance. Another one also examined the effect of MMT on 110 shooters [35]. The duration of intervention lasted for 4 weeks and the results indicated that MMT enhanced sports performance (*p* < 0.001) compared to the control group. In another study of fifty-two university athletes, the participants who received a six-week mindful sport performance enhancement (MSPE) intervention (90 min each week) significantly increased the self-rated sport performance compared to the control condition [36]. In a Portuguese study of twenty-eight elite soccer athletes [38], the results indicated that MBSoccerP was effective in enhancing elite soccer athletes’ performance compared to the control group. In addition, one study used the ACT method to intervene on 34 junior elite ice hockey players and found that ACT had significant effects on both objective performance outcomes (goals, assists, and taken shots) and blinded coach ratings of players performance in comparison with the control condition [57]. Another study with 24 collegiate students found that after the MBI, the participants reported greater sport enjoyment, less negative internal states of current sport performance, and greater improvements in satisfaction with sport performance compared to the PST group [58].

### 3.3. Effects of Mindfulness-Based Interventions on Mindfulness among Athletes

The use of MBIs has yielded significant improvements in mindfulness levels in most studies. For example, two studies of MBSoccerP found that mindfulness intervention was effective in improving the mindfulness level of Portuguese football players [38,39]. One study of BATL found that the MBI was effective in increasing mindfulness in athletes [48]. A Switzerland research group investigated the effectiveness of the MI approach and traditional psychological skills training (PST) in promoting functional athletic behavior in athletes [33]. Elite athletes from 23 different sports were selected (n = 133) and the results indicated that the MI approach could effectively improve the mindfulness level of athletes. In a study of 22 Iranian athletes, the intervention group who received a weekly, seven-session MAC intervention had significantly higher post-test mindfulness scores than pre-test scores. The study also found that increased mindfulness levels were associated with both reduced injury and enhanced performance [40]. In another study of 30 amateur basketball players in Iran, the result found that the experimental group (n =15) had significant higher mindfulness scores than the control group (*p* < 0.05) [51]. In addition, in a study of eight-week MBSR, researchers found that athletes’ pain levels decreased as their level of mindfulness increased, suggesting that mindfulness practice could be an effective adjunct to exercise therapy [53]. Additionally, applying mindfulness exercises in athletes’ daily training could not only increase their mindfulness levels but also decrease the risk of injury [54].

**Figure 3:**
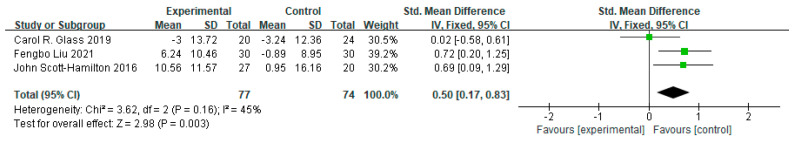
Pooled effect sizes of mindfulness-based interventions on mindfulness among athletes.

Three studies were included in the meta-analysis and a significant moderate effect size was yielded (*d* = 0.50, 95% CI = [0.17, 0.83], *p* = 0.003). There was a from low to moderate heterogeneity among the included studies (I² = 45%, *p* = 0.16). (Figure 3).

### 3.4. Effects of Mindfulness-Based Interventions on Mindfulness-Related Psychological Components among Athletes

For mindfulness-related psychological components, in a Portuguese study, researchers found that MBSoccerP was effective in increasing the attributes of compassion, psychological flexibility, and in which terms that mediates dispositional flow on elite soccer players [39]. MSPE was evident to increase flow and satisfaction with life [38]. Mindfulness-based intervention might be associated with diminished psychological stress responses to competition [37]. In one study of twenty-eight elite soccer athletes, an 8-week MBSoccerP intervention found that the intervention was effective in enhancing participants’ self-compassion, psychological flexibility, and flow [39]. Thirty-two of them improved the performance-relevant psychological factors, especially concerning the handing of emotions and attention control [41]. In Australia, researchers found that MBI tailored to specific athletic pursuits was effective in facilitating flow experiences [42]. A study of 40 elite football players found that the mindfulness meditation sustained the levels of mindfulness skills and attentional control, whereas the control reported decreases in these outcomes during the period of four months [43]. In another MAC intervention, athletes reported that brief mindfulness training could significantly improve athletes flow and resilience and that resilience partly mediated the effect of brief mindfulness training flow [44]. Furthermore, one study showed that 6-week mindfulness training was effective in helping athletes achieve a flow state [47]. In China, one study indicated that reducing pre-sleep arousal and promoting the sleep quality of athletes after night training may be influenced by brief mindfulness induction [50]. Of the last two studies, one of them found that MAC improved dimensions of self-compassion and grit among female adult athletes [55]. Another one suggested that BATL improved both sustained and selective attention in young athletes and that more training in the same amount of time resulted in higher scores in the assessment [56].

**Figure 4:**
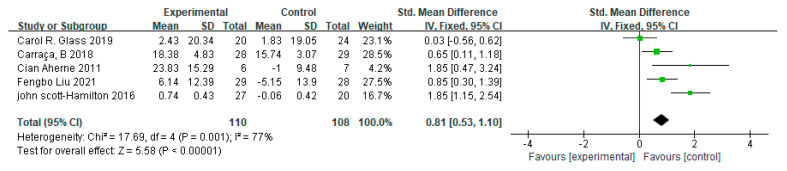
Pooled effect sizes of mindfulness-based interventions on mindfulness-related psychological components among athletes.

Five studies were included in the meta-analysis, showing a large effect size of mindfulness interventions on the psychological components of athletes (*d* = 0.81, 95% CI = [0.53, 1.10], *p* < 0.001). There was high heterogeneity among included studies (I² = 77%, *p* = 0.001) (Figure 4).

### 3.5. Effects of Mindfulness-Based Interventions on Mental Health Indicators among Athletes

Eleven studies examined the effects of MBI on promoting athletes’ mental health. A study of NCAA women’s basketball in USA found that the MAC was an effective intervention for the mental health of female collegiate athletes [13]. A study of retired football players in Iran found that MBSR intervention has the potential to increase their psychological well-being [45]. A RCT study found that elite volleyball athletes who received the MBI significantly reduced their mental fatigue caused by competition compared to the control group [46]. A Norwegian study found that the mindfulness intervention was effective in improving athletes’ burnout [49]. In a USA study, 16 recreational basketball players showed significant reductions in anxiety after a brief mindfulness intervention [29]. Another study from USA found that a brief mindfulness intervention mitigated the effects of ego depletion in a basketball free-throw task [30]. In Iran, one study suggested that a MAC intervention was effective in decreasing athlete’s experiential avoidance and exercise anxiety [31]. Another study using the MAC showed that the MAC approach was a more effective intervention condition in reducing emotion regulation difficulties, as well as enhancing sport-relevant mindfulness skills and perceived athletic training performance in elite sport [32]. Two studies of MMT in India both found that the intervention was effective in decreasing pre-competition stress [34,35]. In addition, one study found that MSPE could significantly reduce worry [36]. In Brazil, after eight gun and pistol shooters underwent MBI intervention, the results showed that mindfulness was effective in reducing pre-competition stress [52].

**Figure 5:**
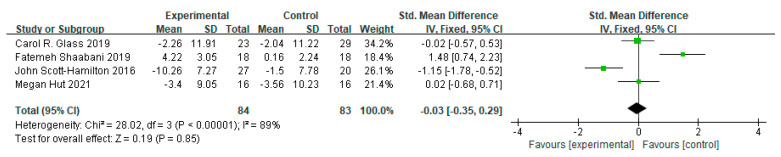
Pooled effect sizes of mindfulness-based interventions on mental health indicators among athletes.

Four studies were included in the meta-analysis (Figure 5), which showed that there was no significant effect of MBI on mental health among athletes (*d* = −0.03, 95% CI = [−0.35, 0.29], *p* = 0.85). There was high heterogeneity among the studies (I² = 89%, *p* < 0.001). 

### 3.6. Publication Bias Assessment, Subgroup Analysis, and Sensitivity Tests

The results of the funnel plots and Egger’s regression tests showed that there was no evidence supporting a significant publication bias of included studies (all *p* > 0.1). For the subgroup analysis, as there was no adequate number of studies (i.e., n < 10) to be included, a further subgroup analysis for the potential moderators was not available. In addition, a sensitivity test was conducted by excluding the studies with a high risk of bias. The results were consistent with primary analyses that included all the studies.

## 4. Discussion

This is the first study that systematically reviewed and meta-analyzed the RCT evidence regarding the effectiveness of MBIs on athletic performance, mindfulness levels, mindfulness-related psychological components, and mental health outcomes among athletes. Thirty-two eligible studies were included in the narrative synthesis, of which nineteen were included in the quantitative analysis. Narratively, the MBIs were effective in promoting athletes’ sports performance. The results of the meta-analysis found that MBIs showed effectiveness on improving the mindfulness and mindfulness-based psychological components, yet no significant effects were found for mental health outcomes among athletes.

For athletic performance, from the narrative synthesis, we found that all the included studies indicated a significant improvement in the performance indicators after receiving the MBI. Our finding is consistent with the previous review papers supporting for the effectiveness of MBIs on athletes’ sport performance [9,12]. However, due to the limited evidence and considerable variety in the outcome measures, a quantitative synthesis is not available. More studies using an RCT design to examine the effects of using MBIs in athletes’ sports performance are needed in the future. 

For mindfulness levels, in accordance with previous evidence [59,60], our study found that MBIs could significantly improve the athletes’ mindfulness levels. For the mindfulness-related psychological components (e.g., acceptance, flow, psychological flexibility, and ruminative response), we found a significant effect of MBIs on improving these indicators, which is in line with previous evidence among different types of athletes and general populations [10,61,62]. During the games, it is easy for athletes to experience negative emotions, such as nervousness and worries [63,64]. On game day, players may experience dissatisfaction with their performance, negative thinking, reality avoidance, and immersion in unpleasant emotions due to off-court circumstances, such as the unfavorable response of the crowd or losing points [65,66]. Mindfulness may play a crucial role in addressing these problems for athletes. Our research findings preliminarily supported the effects of MBIs on improving these mindfulness-related psychological components, implying that, in future practice, MBI could be applied in relevant sports domains to achieve specific training targets (e.g., enhancement of emotion control, prevention of performance failure under pressure, and improvement of flow and commitment).

For the mental health outcomes, our findings were inconsistent with the previous evidence that showed that MBIs were effective in improving mental health symptoms [19,62]. The potential reasons for the discrepancy may be multifaceted. For example, our review targeted all types of athletes, while previous studies focused on the elite athletes or general populations. Another reason MBIs may have not demonstrated improvements in mental health symptoms of athletes is because the athletes in the studies that were selected for our review are not clinical populations. It may have been that athletes’ mental health symptoms were already low; therefore, changes in mental health outcomes may have been inconsequential. In addition, we only included studies using an RCT design, while the previous evidence did not limit the study types for qualitative and quantitative syntheses. Although, in our narrative analysis, most studies supported a significant effect of MBI on athletes’ mental health indicators, the meta-analysis results did not yield a significant effect size. Considering the limited number of studies in the analysis as well as the high heterogeneity, we are not able to provide convincing evidence on the MBI intervention effects on improving the mental health outcomes among athletes, which to some extent emphasize the importance and requirements of more high quality RCTs on this domain.

Several limitations of this review should be noted. First, despite our efforts to implement a thorough literature search in the limited databases, we might omit some studies due to the settings of search strategies (e.g., only limited to English language). Moreover, due to the limited number of studies, we were not able to conduct a subgroup analysis to further identify the moderators (e.g., participants characteristics, MBI types, and outcome measures) that could explain the high degree of heterogeneity. The small number of studies could also result in the cautious interpretation of the research findings. In addition, we did not examine the potential mediators in the MBI programs due to the lack of relevant data such that a further understanding of the underlying mechanisms could not be identified.

Despite these limitations, our research findings add values to future research and interventions on MBIs among athletes. Our findings suggest that MBIs may be a potentially effective approach for improving athletes’ sport performance; however, more strict empirical studies (e.g., RCT and cluster-RCT) should be undertaken to further identify the effectiveness of MBIs and its dose–response effect with athletic performance. Furthermore, MBIs had moderate-to-large effect sizes on enhancing athletes’ mindfulness and mindfulness-related psychological components (e.g., flow, acceptance, and self-compassion), implying that future studies could use MBIs to achieve specific training targets (e.g., enhancement of emotional control and improvement of flow and commitment). In addition, our findings could not provide convincing quantitative evidence for the effectiveness of MBIs on athletes’ mental health outcomes due to the limited number of included studies. More RCT studies focusing on the mental health aspects among athletes are warranted in the future.

## 5. Conclusions

In conclusion, our systematic review and meta-analysis provided preliminary support for the effectiveness of MBIs in promoting athletic performance, mindfulness level, and mindfulness-related psychological components among athletes. The research findings also suggest that more high-quality studies using a rigorous empirical design (e.g., RCT) are warranted in the future, especially with respect to the mental health domains of athletes.

## Figures and Tables

**Table 1:** Characteristics of included studies (n = 32).

Study	Study Design	Population (n)	Age (Mean ± SD)	Duration/Frequency	Measures	Findings
Wolch et al. (2021) [29]	RCT	Male recreational basketball players (n = 32)	21.22 ± 2.01 yrs.	Two sessions	CSAI-2R, TMS, MAAS	A 15 min mindfulness intervention appears to have some effects on participants first basketball free-throw shot and state anxiety when performing under pressure.
Shaabani et al. (2019) [30]	RCT	Experienced male basketball players (n = 72)	28.6 ± 4.0 yrs.	Two sessions	SAS-2, DISE, DSS, PANAS, CHIME, BSCS, EDMC, TMS	A brief mindfulness intervention mitigates the effects of ego depletion in a basketball free-throw task.
Dehghani et al. (2018) [31]	RCT	The women’s basketball team of both Iran and Tehran University of Medical Sciences (n = 31)	23.44 ± 0.49 yrs.; 22.34 ± 0.34 yrs.	Eight sessions	SPQ, AAQ, SCAT	Mindfulness-Acceptance-Commitment-based approach is an effective intervention to increase athletic performance and reduce experiential avoidance and sports anxiety in athletes.
Gross et al. (2018) [13]	RCT	Women’s basketball players from an NCAA Division III university athletic department in the northeastern United States (n = 22)	Nil	7 weeks	CCAPS-62, AAQ-II, SPQ, WAI-S, DERS, MAAS, MC-C	The MAC is an effective intervention for the mental health and sport performance needs of female collegiate athletes.
Josefsson et al. (2019) [32]	RCT	Competitive elite athletes (n = 69)	20.9 ± 4.17 yrs.	7 weeks	AMQ, DERS	MAC approach is a more effective intervention compared to the PST condition in reducing emotion regulation difficulties, as well as enhancing sport-relevant mindfulness skills and perceived athletic training performance in elite sport.
Röthlin et al. (2016) [33]	RCT	Elite athletes from 23 different sports (n = 133)	23.68 ± 6.12 yrs.	Nil	CHIME, CAI-T, Self-generated three-item measure	Trait mindfulness is related to fewer performance worries and prevents the remaining worries from influencing athletes’ behavior, thereby helping them to perform better.
John et al. (2011) [34]	RCT	Male elite level shooters (n = 96)	29.5 ± 4.3 yrs.	4 weeks	Measure of shooting accuracy or shooting score	MMT may decrease PCS and will enhance PS. It is concluded that 4 weeks of MMT has an effect on HPA-Axis by decreasing the level of SC as a reliable physiological marker of PCS.
Kachanathu et al. (2013) [35]	RCT	Male elite level shooters (n = 110)	29.5 ± 4 yrs.	4 weeks	Measure of shooting accuracy	MMT may decrease PCS and therefore enhance SP.
Glass et al. (2019) [36]	RCT	University athletes (n = 52)	19.32 ± 1.25 yrs.	6 weeks	DASS-21, SWLS, FFMQ, AAQ-II, DFS-2, SAS, Mindfulness practice summary, SRF, CRF	Significant increases in flow, trait mindfulness, satisfaction with life, and self-rated sport performance, along with reductions in worry.
Röthlin et al. (2016) [33]	RCT	Elite, sub-elite, and recreational athletes (n = 108)	Nil	5 weeks	CAI-S, TOQS, TOPS, FFMQ-SF, AMQ, SEC-27, AAQ-II, EQ, DSS, ANT, TEOSQ, RSC, SCS, and BSI-18	Both PST and MBI are expected to help improve functional behavior in athletes.
Mehrsafar et al. (2019) [37]	RCT	Elite Wushu athletes (n = 26)	25.4 ± 2.4 yrs.	8 weeks	CSAI-2R and MAAS	Mindfulness-based intervention might be associated with a diminished physiological and psychological stress responses to competition.
Carraça et al. (2019) [38]	RCT	Elite soccer athletes (n = 57)	25.79 ± 3.3 yrs.	8 weeks	AAQ-II, FFMQ, DFS-2, SCS, WBSI, BSI, Athlete’s—FAIP-A	MBSoccerP can be effective in enhancing elite soccer performance, self-compassion, psychological flexibility, mindfulness, and flow.
Carraça et al. (2019) [39]	RCT	Elite soccer athletes (n = 57)	25.79 ± 3.3 yrs.	8 weeks	AAQ-II, FFMQ, DFS-2, SCS, WBSI, BSI, Athlete’s—FAIP-A	Mindfulness-Based Soccer Program (MBSoccerP) is effective in increasing the attributes of mindfulness, self-compassion, and psychological flexibility and in terms that mediate the dispositional flow and peak performance on elite soccer players.
Mozafari Zadeh et al. (2019) [40]	RCT	Amateur soccer players (n = 44)	24.15 (24.86 ± 4.68) (23.77 ± 1.95) yrs.	Seven sessions	Mindful Sport Performance Questionnaire, 7-point Likert scale (individual and team performance) and injury occurrence and severity	Mindfulness training shows promise in preventing injury and improving performance.
Röthlin et al. (2020) [41]	RCT	Athletes from four sports (n = 95)	24.43 ± 5.15 yrs.	4 weeks	FFMQ-SF, TOPS, AAQ-II, SEC-27, TOQS, EQ, and ASOAF6	Both forms of mental training led to improvements in performance-relevant psychological factors, especially concerning the handling of emotions and attention control.
Scott-Hamilton et al. (2016) [42]	RCT	Cyclists (n = 47)	39.81 (38.96 ± 12.4; 40.65 ± 10.88) yrs.	8 weeks	DFS-2, SAS-2, FFMQ, SASS	Mindfulness-based interventions tailored to specific athletic pursuits can be effective in facilitating flow experiences.
Baltar et al. (2018) [43]	RCT	Elite football players (n = 40)	23.6 ± 1.4 yrs.	12 weeks	ACS, KIMSs-Short	Mindfulness meditation does not improve attentional control or mindfulness skills; however, it prevents those variables from showing decreases among elite football players.
Liu et al. (2021) [44]	RCT	University athletes (n = 60)	19.7 (19.9 ± 0.7; 19.5 ± 0.8) yrs.	Once	FFMQ, DFS-2, and RISC	Brief mindfulness training could significantly improve athletes flow and resilience; resilience partly mediated the effects of brief mindfulness training on flow.
Norouzi et al. (2020) [45]	RCT	Retired football players (n = 40)	34.05 ± 1.72 yrs.	8 weeks	PSS, BAI, MADRS, RSPWB	MBSR intervention has the potential to reduce symptoms of stress, anxiety, and depression and to increase their psychological well-being.
Coimbra et al. (2021) [46]	RCT	Elite female volleyball athletes (n = 30)	<18 yrs.	2 weeks	Total Quality Recovery scale, Mental Fatigue Visual Analog Scale, Physical Fatigue Visual Analog Scale	The mindfulness intervention effectively attenuated the mental fatigue caused by competition in volleyball athletes.
Aherne et al. (2011) [47]	RCT	Athletes from a University High Performance Centre (n = 13)	21.00 ± 1.68 yrs.	6 weeks	DFS-2, CAMS-R	Mindfulness training appears to be an appropriate method to help athletes to achieve a flow state and, therefore, seems likely to be an effective performance enhancement strategy as well.
Jekauc et al. (2016) [48]	RCT	Students in sport science of Humboldt University Berlin (n = 46)	23.4 ± 4.1 yrs.	8 weeks	MAAS	BATL is an effective strategy to increase mindfulness in athletes.
Moen et al. (2015) [49]	RCT	Norwegian junior athletes in sports (n = 77)	18 yrs.	12 weeks	MAAS, PSS-14, ABQ, ASQ	Significant effects from the mindfulness intervention on athlete burnout. There were no significant effects found on perceived stress or perceived performance in school and sports.
Li et al. (2018) [50]	RCT	University athletes (n = 63)	21.16 ± 1.79 yrs.	Once	PSQI, Rating Perceived Exertion Scale, PSAS, Five-item Chinese-translated State Version of the Mindful Attention Awareness Scale, Sleep and Health Research Laboratory’s Sleep Diary	The brief mindfulness induction may be an effective approach for decreasing pre-sleep arousal and improving sleep quality after night training among athletes.
Ajilchi et al. (2019) [51]	RCT	Male amateur basketball players (n = 30)	22–24 yrs.	6 weeks	Mindful Sport Performance Questionnaire, MT, SEIS	These findings may have implications on sport mindfulness training in increasing the MT and emotional intelligence of athletes.
Samadi et al. (2021) [52]	RCT	Male shooters (n = 24)	17–22 yrs.	6 weeks	Cortisol with ELISA method	The practice of psychological skills, especially mindfulness, can be used to reduce pre-competition stress.
Bagheri et al. (2021) [53]	RCT	Female recreational runners with patellofemoral Pain (n = 30)	28.3 ± 7.08 yrs.	8 weeks	Pain intensity, Knee Symptoms and Function, Global Rating of Change Scale, Tampa Scale of Kinesiophobia, Pain Catastrophizing Scale, and Coping Strategies Questionnaire	Mindfulness practice can be an effective adjunct to exercise therapy in the rehabilitation of PFP in recreational female runners.
Ivarsson et al. (2015) [54]	RCT	Junior elite soccer players (n = 41)	16.97 ± 0.79 yrs.	7 weeks	Nil	Applying mindfulness exercises in athletes daily training to help lower injury risk.
Mohebi et al. (2021) [55]	RCT	Female athletes at national competition level (n = 40)	22.22 ± 2.40 yrs.	7 weeks	MIS, SCS-SF, and SG-S	While the active control condition improved dimensions of mindfulness, self-compassion, and grit among female adult athletes, improvements were much stronger in the Mindfulness Acceptance Commitment condition.
Kittler et al. (2022) [56]	RCT	Pupils of a German elite sports school (n = 137)	12.23 ± 0.50 yrs.	6 weeks	FAIR-2	The results of this study indicate that the Berlin Mindfulness-Based Training for Athletes (BATL) improved both sustained and selective attention in young athletes and that more training in the same amount of time resulted in higher scores in the assessment.
Lundgren et al. (2022) [57]	RCT	Junior elite ice hockey players (n = 34)	18.09 ± 0.88 yrs.	4 weeks	Coach Ratings, Version of the credibility questionnaire	Significant effects on both objective performance outcomes (goals, assists, and taken shots) and blinded coach ratings of players performance, focus, and commitment to their development in favor of the ACT group.
Hut et al. (2021) [58]	RCT	Members of an NCAA Division III Track and Field team (n = 32)	19.52 yrs.	6 weeks	SDFS-2, CDFS-2, DASS-21, SAS-2, PHLMS, MIS, DERS-SF, BEAQ, SRF, PEQ	The 24 collegiate athletes who completed post-test measures showed significant improvements in sport anxiety and reported greater sport enjoyment and less influence of negative internal states on current sport performance; the MSPE group reported greater improvements in satisfaction with sport performance compared to PST.

Note. RCT = randomized controlled trial; SD = standard deviation; MAC = Mindfulness-Acceptance-Commitment; PST = psychological skill training; MMT = mindfulness meditation therapy; PCS = Pre-Competition Stress; PS = Performance of Shooting; HPA: Hypothalamic Pituitary Adrenal; SC = Salivary Cortisol; MBI = mindfulness-based intervention; MBSR = mindfulness-based stress reduction; MSPE = mindful sport performance enhancement; CSAI-2R = Competitive Sport Anxiety Inventory-II Revised; TMS = Toronto Mindfulness Scale; MAAS = Mindful Attention Awareness Scale; SAS-2 = Sport Anxiety Scale-2; DISE = Daily Inventory of Stressful Events; DSS = Depletion Sensitivity Scale; CHIME = Comprehensive Inventory of Mindfulness Experiences; BSCS = Brief Self-Control Scale; EDMC = Ego-Depletion Manipulation Check; CAI-T = Competition Anxiety Inventory Trait; DASS-21 = Depression, Anxiety, and Stress Scales-21; SWLS = Satisfaction with Life Scale; FFMQ = Five-Facet Mindfulness Questionnaire; AAQ-II = Acceptance and Action Questionnaire-II; DFS-2 = Dispositional Flow Scale; CRF = Coach’s Rating Form; SRF = Sport Rating Form; TOPS = Test of Performance Strategies; SEC-27 = Self-Assessment of Emotional Competencies; TOQS = Thought Occurrence Questionnaire for Sport; EQ = Experience Questionnaire; ASOAF= Action orientation after failure and state orientation after failure; DFS-2 = Dispositional Flow Scale-2; SASS = Sport Attribution Style Scale; PSS = Perceived Stress Scale; MADRS = Montgomery-Asberg Depression Rating Scale; RSPWB = Ryff Scales of Psychological Well-Being; PSQI = Pittsburgh Sleep Quality Index; PSAS = Pre-sleep Arousal Scale; FAIR-2 = Frankfurt Attention Inventory-2; SDFS = Short Dispositional Flow Scale; CDFS-2 = Core Dispositional Flow Scale-2; PHLMS = Philadelphia Mindfulness Scale; DERS-SF = Difficulties in Emotion Regulation Scale-Short Form; BEAQ = Brief Experiential Avoidance Questionnaire; and PEQ = Program Evaluation Questionnaire.

## Data Availability

Data is available by contacting the corresponding or first authors.

## Supplementary Materials

The following supporting information can be downloaded at: https://www.mdpi.com/article/10.3390/ijerph20032038/s1. Table S1: PRISMA 2020 Checklist.
